# Using neural networks to support high-quality evidence mapping

**DOI:** 10.1186/s12859-021-04396-x

**Published:** 2021-10-21

**Authors:** Thomas B. Røst, Laura Slaughter, Øystein Nytrø, Ashley E. Muller, Gunn E. Vist

**Affiliations:** 1 Department of Computer Science, Norwegian University of Science and Technology (NTNU), Trondheim, Norway; 2 Reviews and Health Technology Assessments, Norwegian Institute of Public Health (NIPH), Oslo, Norway

**Keywords:** Evidence maps, Evidence based medicine, Knowledge dissemination, Automated coding, Machine learning, Deep learning

## Abstract

**Background:**

The Living Evidence Map Project at the Norwegian Institute of Public Health (NIPH) gives an updated overview of research results and publications. As part of NIPH’s mandate to inform evidence-based infection prevention, control and treatment, a large group of experts are continously monitoring, assessing, coding and summarising new COVID-19 publications. Screening tools, coding practice and workflow are incrementally improved, but remain largely manual.

**Results:**

This paper describes how deep learning methods have been employed to learn classification and coding from the steadily growing NIPH COVID-19 dashboard data, so as to aid manual classification, screening and preprocessing of the rapidly growing influx of new papers on the subject. Our main objective is to make manual screening scalable through semi-automation, while ensuring high-quality Evidence Map content.

**Conclusions:**

We report early results on classifying publication topic and type from titles and abstracts, showing that even simple neural network architectures and text representations can yield acceptable performance.

## Background

Experts, policy makers and researchers worldwide are scrambling to keep up with the influx of potentially relevant COVID-19 studies. Research is being published at an unprecedented pace and in volumes never seen before. Whereas a traditional peer review- and journal-based publication process would take 6–12 months, research findings now often find their way to readers in a matter of days or weeks. The use of preprint servers, with only cursory quality checks, is increasing. While this has had a positive impact on knowledge dissemination speed in the medical sciences, this arguably comes at a cost to quality, reliability and trustworthiness [1].

The need for timely, informed and quality-assessed knowledge is widely recognised as crucial for handling the ongoing COVID-19 pandemic. One initiative to meet this need, known as the Living Evidence Map Project, was launched at the Norwegian Institute of Public Health (NIPH) within their Division for Health Services [2]. The NIPH has a large team of experienced review authors that regularly conducts systematic reviews of medical science research, this as part of their mandate to inform evidence-based decisions pertaining to prevention and infection control. Evidence maps provide a useful overview of the literature, but since many of the workflow steps overlap, they can be seen as a precursor towards the production of systematic reviews.

The challenge with the current approach to evidence mapping is that it is currently mostly manual and requires considerable amounts of expertise from the reviewers. This leads to a review and coding process that is already struggling to keep up with the volume of incoming publications and that is hard and costly to scale. We believe that technologies of medical language processing, knowledge extraction and machine learning have the potential to assist and amplify the expertise required to produce systematic reviews and evidence maps.

Automation was not introduced to synthesising medical evidence in the past since it was thought to be inadequate and would potentially only increase the amount of work needed, adding effort and time to check over machine results. We began with exploring past work that assessed the use of text mining to support systematic review workflows. Projects from years prior to COVID focused on the literature screening phase of the work process and some have been implemented in the current reviewing support systems [3].

Although the screening tools have been implemented into workflows, NIPH has no automated support that would speed up coding procedures. We have initiated a series of experiments to explore multi-label deep learning classification to help with this task. In this work, we conduct four experiments in order to assess the possibilities of using deep learning techniques in the evidence mapping workflow. Automated approaches are evaluated based on measurements of precision and recall, however, we know very little about what this means to those who wish to implement automation into workflows where a high-quality knowledge product is expected as the result. We hope to learn more about acceptable error rates when applied to a real-life needs and workflows. Therefore, our research is centred around the question of whether it is possible to reduce manual efforts while at the same time maintaining high-quality evidence maps.

We received a set of training data from NIPH as a result of their manual coding to produce evidence maps for COVID-19, and we focused our research questions on exploring the classification of publications for automated coding. Our main research question centres on performance when using deep learning models to classify the COVID-19 research literature: *Can we expect an accurate classification of clinical research topics, publication type, and data types using only publication titles and abstracts?*

We believe that this work will provide the groundwork for understanding the implementation of machine learning and deep learning techniques in real-life clinical scenarios and workflows. The Allen Institute for Artificial Intelligence put forth the CORD-19 dataset [4] which is a set of scientific publications available related to COVID-19 as well as related historical coronavirus research, including SARS and MERS. We make use of this dataset in our experiments, and seek to tie the challenges outlined to the needs related to evidence mapping and generation of systematic reviews.

### The NIPH coding workflow

In the NIPH coding workflow, incoming articles that will be coded for inclusion in the evidence maps have been screened and quality controlled. In this context the term “coding” means manual classification of each article according to a predefined set of discrete categories, e.g. paper topic (diagnosis, etiology etc.) or data type (primary data, secondary data etc.), some of which will be discussed in more detail later. The articles include those that their collaborator, EPPI-Centre [5], has already screened (using a combination of machine learning and manual methods.) These are supplemented with studies from NIPH’s own searches. As of 15 July 2020, the map contains 6513 publications categorised by topic, population, and publication type.

The categorisation process is labour-intensive. Depending on the study, it may take 3–15 min to code a paper. Studies from the corpus are randomly allocated to two coders, a “core coder” and an “external coder”. The core coder has the ability to see the external coder’s coding. When the external coder codes first, the core coder can see those codes while coding themselves. The process is manual and is done to increase the speed of work for the core coder who has greater expertise.

The breadth of studies brings with it many publication types and topics that are not always easy to categorize. In the case of disagreement between coders, differences are discussed in a reconciliation meeting. This process usually means that the core coder’s codes are adopted as the final version, and sometimes with some extra codes added after input from the external. This is on-the-ground learning for the external coder, because it’s really the only time they see how a study “should” be coded. The benefit of this process is a continuous overview over the consistency in the coding efforts. Differences in coding may be due to different viewpoints or human error, but were mostly resolved without the need of a third researcher to adjudicate.

NIPH has 28 coders in total, all with a research or medical background. There are 15 external volunteers and 13 from the Norwegian Institute of Public Health. There is a programme to train new coders with the coding process, with an introduction to the necessary software and the NIPH coding manual. Following the training, new coders are then paired up with an experienced coder to continue training on-the-job, with weekly discussions for general questions or specific studies. During these discussion rounds, any disagreements in coding are discussed and reconciled. Currently coders are managing to code over 10 studies per hour.

NIPH has created its own coding system with an accompanying manual describing all codes in detail, this with the aim of reducing ambiguity. The NIPH coding manual has developed throughout the project to address the developing research. This dynamic approach has allowed for much-needed flexibility, but at times this can require considerable work to realign older codes.

### Related work

Deep learning machine learning models are seeing increased use for a wide variety of natural language processing (NLP) tasks [6], motivated by the ability to produce results that improve on the performance of previous-generation machine learning methods. For text classification, they have surpassed traditional methods for tasks such as sentiment analysis, news categorization and natural language inference [7]. In the medical domain, there has been a great deal of focus on deep learning for medical image processing [8] but other avenues of research are continuously opening up. For example, it has been shown to perform well for identifying relevant publications from medical literature, especially when considering that less time is spent on e.g. feature engineering and MeSH term linking [9]. Convolutional neural networks, a particular deep learning architecture, have shown promising performance for tasks such as automated ICD-9 coding [10] and de-identification of clinical texts [11]. A recent study concluded that the use of deep learning methods has yet to fully penetrate clinical natural language processing but also that usage was increasing rapidly [12].

Production of evidence maps, systematic reviews as well as best practice guidelines have been discussed in terms of the ecosystem of healthcare system data. Connecting and reusing health data is an essential aspect to implementing precision medicine. The flow of data from patient care and clinical trials to published results and observations, and through the cycle of summarization and reuse to inform care has also been connected to the concept of learning healthcare systems. Even prior to the COVID-19 crisis, the issues and problems have been identified as evolving and cutting-edge research [13]. Recently published discussions on evidence ecosystems call for more coordinated and integrated synthesis that is relevant, trustworthy, and useful for decision making [14].

Recent publications and work from 2016 until the present time has originated from the group at the National Centre for Text Mining, University of Manchester. They focused their research on the literature screening phase of the systematic review process, with methods developed for prioritizing references [15], document clustering using a predictive network [16], and topic detection [17]. In addition, they built a prototype based on the sum of their work, Robot Analyst. The work was completed as part of a funded project titled Supporting Evidence-based Public Health Interventions using Text Mining with collaboration of the University of Liverpool Machine Learning and Data Analytics group, and the National Institute for Health and Care Excellence (NICE). This group was influential and together with the EPPI systematic review tool developers at UCL, EPPI implemented screening functionality into their production system.

There are several other examples of research on reducing the manual effort associated with classification of scientific literature. A 2006 study by Cohen et al. used machine learning for automated classification of document citations, this with the purpose of aiding experts in updating system reviews of drug class efficacy [18]. Moving beyone the medical domain, work has been done on e.g. classification of mathematical research [19] and on general research literature with the purpose of applying the correct Dewey Decimal Classification code [20]. While much work focuses on classification of English-language literature, examples of using machine learning methods for automated coding of scientific literature in the Russian language [21]. Most approaches appear to be based on supervised learning but use of unsupervised learning also exists [20].

## Methods

The coding data was exported as a JSON file on May 4th, 2020, from the EPPI-Mapper [22] tool used by NIPH. It had two main sections, CodeSets and References, containing respectively the coding definitions and the publications with the applied codes. To get a better feel for the type and volume of data available to us we analyzed the data coverage in the CodeSets and References sections.

The codes in the CodeSets section has a tree structure where each attribute node has an ID, a name, a description, a set ID, a set description and a type. Parent nodes also has a list of child attributes. In total there are 40 parent attributes and 223 leaf attributes.

**Table 1:** Reference field coverage

Key	# References	Cov. (%)
Abstract	1151	86.41
Authors	1321	99.17
Availability	0	0.00
City	9	0.68
Codes	1332	100.00
Comments	689	51.73
Country	0	0.00
CreatedBy	1324	99.40
DOI	1216	91.29
DateCreated	1332	100.00
DateEdited	1332	100.00
EditedBy	1332	100.00
Edition	0	0.00
Institution	464	34.83
Issue	316	23.72
ItemId	1332	100.00
ItemStatus	1332	100.00
ItemStatusTooltip	1332	100.00
Keywords	446	33.48
Month	1	0.08
OldItemId	1332	100.00
Outcomes	0	0.00
Pages	513	38.51
ParentAuthors	0	0.00
ParentTitle	1331	99.92
Publisher	8	0.60
ShortTitle	1332	100.00
StandardNumber	506	37.99
Title	1332	100.00
TypeName	1332	100.00
URL	943	70.80
Volume	449	33.71
Year	1332	100.00

There was a total of 1332 references. Each reference section had a number of metadata fields. Some of these, such as *Abstract*, *Authors* and *Volume* were directly related to the associated publication. The rest of the fields, such as *Codes* and *Comments* contained data that had been added by the coders during the publication coding process. Table 1 shows an overview of how well these fields are covered in the dataset. We see that *Codes* coverage is complete, as could be expected, while *Comments* and *Keywords* are more sparsely used. As for bibliographic data, the publication *Title* is always present while some *Abstract* entries are missing. The journal or conference title is found in the *ParentTitle* field which is absent in only 1 case. The *ItemId* field was confirmed to be unique. The *Keywords* value, if available, contains a newline separated list of coder-provided keywords. In conversations with NIPH we learned that their coders did not add keywords and that the origin of this information is therefore of uncertain quality.

**Table 2:** Matching FHI data with CORD-19 data

Match element	Matches	Matches w/ abst.
Title	123	28
DOI	127	33

For 181 reference entries the abstract is missing, which means that very little textual data beyond the title, keywords and comments is available as classification features. To alleviate this we attempted to link the references without abstracts to the COVID-19 Open Research Dataset (CORD-19) [4], specificially to the main metadata.csv file, using publication title and DOI as linking identifiers. The results can be seen in Table 2, showing that matching on DOI performed the best but even then only 33 missing abstracts could be found. We did not do any normalization on the link values apart from converting to lower case so it is possible that some links were missed this way. It is also possible that in many cases abstracts are simply not available. We decided to augment the data used for the experiments with the additional abstracts found from DOI matching so as to maximize the amount of data available to us.

**Fig. 1:**
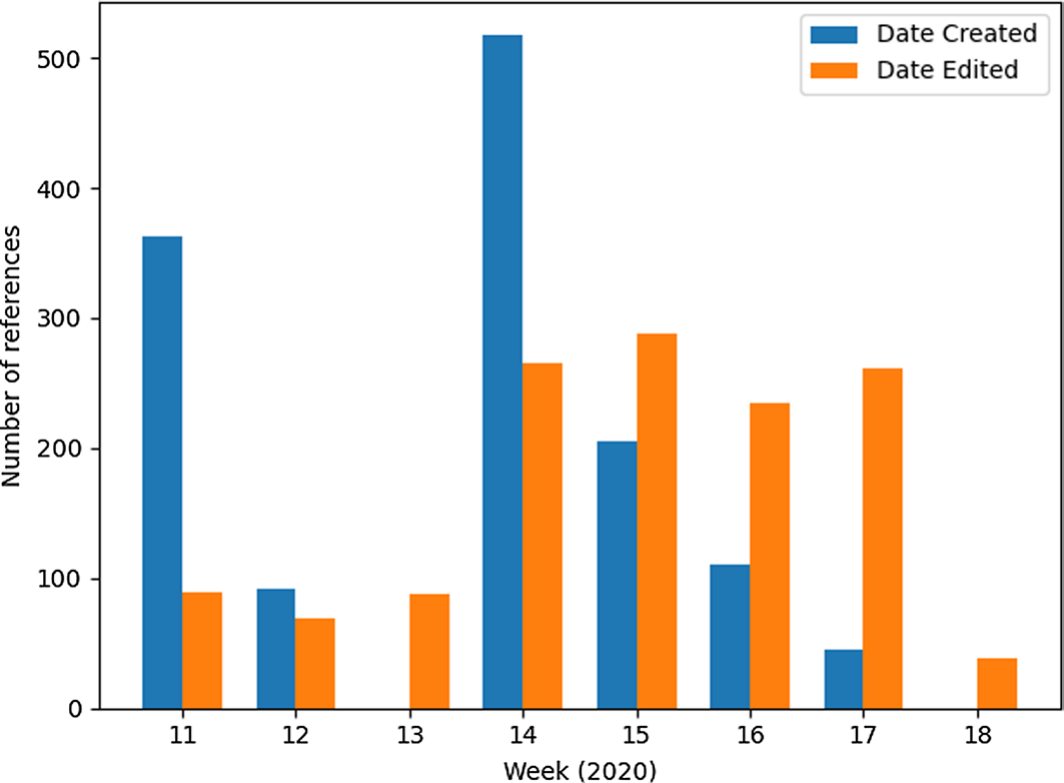
Coded references per week

To get a feel for how the coding practice has evolved we looked closer at the *DateCreated* (when a reference was imported into the system) and *DateEdited* (when a reference was coded) fields. Figure 1 shows how many references were imported and coded per week for the duration of the data sets. The number of coded references increase towards the latter half of the period. The team started with 4 coders and by week 14 this number had increased to 10, the majority of whom were part-time coders. Note that using this field to assess initial coding time is not entirely accurate as sometimes the post-coding quality control process would lead to a reference being recoded. There is also a chance that any administrative changes to other fields could impact the value of the *DateEdited* field.

We then looked at how the actual codes were distributed across the code hierarchy. Many of the codes had an associated *AdditionalText* field with comments made by the coder; coders were instructed to use this field to flag things for discussion during the coding reconciliation process. Other than that there was no additional metadata. Table 3 shows some key numbers regarding code use. Several of the code attribute IDs could not be found in the code set. For some reason some of the coded attribute IDs mapped to the attribute set IDs rather than the standard attribute IDs; this needs to be investigated further.

**Table 3:** Coding statistics

Number of codes	25,133
Avg. number of codes per ref.	18.9
Number of comments	265
Number of unknown codes	1234
Number of unique unknown codes	25

For each code found in a reference we stored the reference ID so as to know how many references are available for a given code. Table 4 shows an overview of the top-level codes and for how many references in the full data set these codes have been applied, sorted by the number of coded references. Codes used only once or never are not shown. We see that data coverage is incomplete for all but the top 5 top-level codes. The more specific topic codes in the bottom half of the table contained more detailed drilldown into the various subtopics. In our experiments we ended up focusing on the *Topic*, *Data Type* and *Population* codes, the main reasons being that they all had single-level coding hierarchies and reasonably well distributed classes.

**Table 4:** Root code use

Class	# Refs
Publication type, detailed	1332
Publication type	1332
Data type	1332
Population	1332
Topic	1332
Topic: Diagnosis	454
Topic: Aetiology	452
Topic: Prognosis	424
Topic: Prevalence	206
Topic: Interventions to treat the infected patient	162
Topic: Interventions targeted at system level to improve management of the pandemic	143
Topic: Experiences and perceptions; consequences; social, political, economic aspects	126
Topic: Infection prevention and control	119

## Results

For our experiments we made a selection of codes that we thought would be best suited as classification labels, with the selection criteria being the amount of data and overall class balance. All experiments were run using the Keras deep learning framework [23]. Keras was chosen because it is a popular and increasingly used framework which also provides several convenience functions to lessen the workload both for text pre-processing and the general machine learning experiment workflow. We relied on the default TensorFlow [24] symbolic math library backend. The choice of deep learning machine learning methods is not only motivated by recent performance advances but also because they usually reduce the need for activities such as feature engineering [25]. For the rest of the discussion, we will refer to codes as labels given that this is a more common terminology for classification tasks.

### Experiment 1: classifying topics from the publication title

**Table 5:** Reference count (Topic)

Class	# Refs
Topic	1332
Prevalence and incidence	205
Etiology	452
Diagnosis	454
Infection prevention and control	119
Interventions to treat the infected patient	162
Interventions targeted at system level	142
Prognosis	424
Experiences and perceptions; consequences; social, political, economic aspects	126

For our initial experiment we wanted to build a classifier for the *Topic* label, as shown in Table 5. The goal is to correctly classify the topic based on information available to us, such as the publication title, the abstract, the publication outlet and so on. The total number of applied labels is 2084. Since this exceeds the number of references it follows that some of the references must be labelled with multiple topics, making this a multi-label classification task. While some topics occur more often than others, there are no topics that are exceptionally scarce and the dataset is relatively balanced.

We first attempted to use only the publication title as input for our features. The primary benefit of using the title is that we know that it always will be present in the dataset. We put all titles into the Keras Tokenizer API, which splits on whitespace, removes punctuation, lowercases all tokens and outputs a bag-of-words-encoded feature matrix with a selected output mode. Each row in the matrix has a vector with the size of the vocabulary, with each word having its own position. In our case we relied on the count mode which means that the word frequency is used as a feature value. For all experiments we set a maximum vocabulary size of the 1,000 most frequent words in the corpus.

We decided to start with the simplest possible neural network architecture, using a sequential model with three dense layers, each having 64 units. The number of layers was set after some initial experimentation to make sure that the model would not be lacking in representational power. The number of layers and units was motivated by similar classification examples as described by the creator of Keras [25] rather than previous experience. Each layer used the relu activation function. The fourth and final layer had 8 units, corresponding to the number of classes, and a sigmoid activiation function. As per recent best practices for this type of classification task we used the Adam optimization algorithm, a binary_crossentropy loss function and, since this was a multi-label classification problem, the categorical_accuracy evaluation metric. We also added precision and recall metrics as these would be more useful in practice for evaluation purposes. Batch size was set to 128. Since we had a fairly small amount of data to work with we used 4-fold cross validation for all experiments, averaging the results. The number of epochs was set by doing trial runs with 20% of the training data set aside for validation. We then observed the loss function output and chose the final number of epochs to roughly correspond with the loss function minimum, this to avoid overfitting. For the final run we used all available data for training and ignored validation. When evaluating on the data set aside for testing we would end up with a vector of values between 0.0 and 1.0. If the value was above a threshold of 0.5 we interpreted this as a positive prediction for the given class.

Table 6 shows the classification results for the *Topic* label in the form of average precision, recall and F1 metrics as well as the standard deviation. We see that precision is in general better than recall, while recall seems to be positively correlated with the amount of training data. For the classes with less data the precision standard deviation is high and results would fluctuate considerably between each run. The difference between precision and recall performance could be explained by the lack of data in the titles: the model picks up on commonly occurring words which makes for safe predictions while the majority of titles have too little information to make a good prediction.

**Table 6:** Topic classification results from title

Class	Precision	Recall	F1
Diagnosis	0.71 (0.06)	0.61 (0.08)	0.66 (0.07)
Etiology	0.69 (0.04)	0.52 (0.08)	0.59 (0.04)
Experiences and perceptions; consequences; social, political, economic aspects	0.80 (0.10)	0.38 (0.07)	0.51 (0.07)
Infection prevention and control	0.72 (0.23)	0.11 (0.02)	0.18 (0.03)
Interventions targeted at system level	0.23 (0.27)	0.08 (0.12)	0.11 (0.17)
Interventions to treat the infected patient	0.73 (0.06)	0.39 (0.04)	0.51 (0.04)
Prevalence and incidence	0.63 (0.07)	0.28 (0.03)	0.39 (0.04)
Prognosis	0.73 (0.05)	0.61 (0.04)	0.66 (0.02)

### Experiment 2: classifying topics from the publication abstract

For the next experiment we stuck with the *Topic* coding from experiment 1 but switched the input data source from the title to the abstract. This would presumably give the deep learning model more data to work with. We kept all other tokenization parameters and model hyperparameters equal, including the network architecture.

Results from classifying the *Topic* label based on abstracts are found in Table 7. The most noticeable difference is that both precision and recall have improved for the classes that performed poorly in the previous experiment. The abstract will in most cases be substantially longer than the title and as such there is more information to work with for the neural network model, thus improved performance is as expected.

**Table 7:** Topic classification results from abstract

Class	Precision	Recall	F1
Diagnosis	0.72 (0.09)	0.68 (0.06)	0.70 (0.03)
Etiology	0.69 (0.06)	0.50 (0.04)	0.58 (0.04)
Experiences and perceptions; consequences; social, political, economic aspects	0.79 (0.08)	0.45 (0.05)	0.57 (0.06)
Infection prevention and control	0.77 (0.13)	0.21 (0.05)	0.33 (0.07)
Interventions targeted at system level	0.65 (0.21)	0.15 (0.07)	0.23 (0.10)
Interventions to treat the infected patient	0.75 (0.08)	0.38 (0.04)	0.50 (0.04)
Prevalence and incidence	0.71 (0.06)	0.30 (0.06)	0.42 (0.05)
Prognosis	0.74 (0.02)	0.56 (0.06)	0.63 (0.03)

### Experiment 3: classifying publication type from the publication abstract

We repeated the same experiment, using the abstracts as a basis for our features but this time attempting to classify for the *Publication type* label. The class distribution can be seen in Table 8. As before, this is a multi-label classification task but this time publications are much more likely to have a single label applied.

**Table 8:** Reference count (Publication type)

Class	# Refs
Publication type	1332
Systematic reviews	156
Studies and modelling	1051
Non-systematic reviews and others	194

With all parameters from the previous experiments kept equal the results are shown in Table 9. Performance for the *Studies and modelling* class is best but this is also by far the most prevalent class.

**Table 9:** Publication type classification results from abstract

Class	Precision	Recall	F1
Non-systematic reviews and others	0.52 (0.41)	0.05 (0.06)	0.08 (0.11)
Studies and modelling	0.86 (0.02)	0.98 (0.01)	0.92 (0.01)
Systematic reviews	0.91 (0.07)	0.53 (0.08)	0.67 (0.07)

### Experiment 4: classifying data type from the publication abstract

This experiment was again similar to the previous one but now for the *Data type* label. This label says something about the kind of data, if any, that was used in the publication. Table 10 shows the class distribution and that most of the reviewed publications deal with primary data.

**Table 10:** Reference count (Data type)

Class	# Refs
Data type	1332
Primary data	789
Secondary data	231
Modelled/computed	271
No data (i.e. comment, editorial)	74

Results of this experiment can be seen in Table 11.

**Table 11:** Data type classification results from abstract

Class	Precision	Recall	F1
Modelled/computed	0.77 (0.09)	0.66 (0.05)	0.71 (0.04)
No data (i.e. comment, editorial)	0.00 (0.00)	0.00 (0.00)	0.00 (0.00)
Primary data	0.80 (0.02)	0.92 (0.02)	0.86 (0.01)
Secondary data	0.89 (0.04)	0.50 (0.01)	0.64 (0.01)

### Experiment 5: classifying topics from the publication abstract with CNN and pre-trained word embeddings

The final experiment is a repeat of experiment 2 but this time with a more advanced architecture which is also supported by pre-trained word embeddings. We used the 100-dimensional GloVe embeddings of 400K words which is based on data from Wikipedia [26]. Individual words were mapped to known embeddings and then fed into an embedding layer. We also used the Keras Bidirectional, GRU, Conv1D, GlobalAveragePooling1D and GlobalMaxPooling1D layers, effectively implementing a bidirectional recurrent neural network.

Results from classifying the *Topic* label based on abstracts with this alternative architecture can be seen in Table 12. When compared with experiment 2 results are either equal or slightly worse. It is reasonable to assume that the lack of training data is a contributing factor.

**Table 12:** Topic classification results from abstract (bidirectional RNN)

Class	Precision	Recall	F1
Diagnosis	0.78 (0.05)	0.58 (0.03)	0.67 (0.01)
Etiology	0.70 (0.03)	0.47 (0.06)	0.56 (0.03)
Experiences and perceptions; consequences; social, political, economic aspects	0.77 (0.11)	0.40 (0.05)	0.51 (0.02)
Infection prevention and control	0.53 (0.09)	0.19 (0.06)	0.28 (0.08)
Interventions targeted at system level	0.59 (0.07)	0.11 (0.08)	0.16 (0.12)
Interventions to treat the infected patient	0.76 (0.05)	0.29 (0.04)	0.41 (0.03)
Prevalence and incidence	0.54 (0.00)	0.28 (0.17)	0.34 (0.15)
Prognosis	0.67 (0.02)	0.55 (0.05)	0.61 (0.02)

## Discussion

A common issue with all experiments was lack of labeled data, which would definitely impact classification performance. Also, we only used the title or the abstract for features, which would impact some of the experiments. Beyond sparse data we know from conversations with NIPH that some of the *Topic* labels are impossible to infer from the publication title alone, which may explain the performance improvement in experiment 2 where the abstract was used instead of the title. At the same time, some of the other classes see no—or even a negative—boost to performance. The simple bag-of-words representation may be partly at fault. In addition, for some publications no abstract is available, even after augmenting with additional abstracts from the CORD-19 dataset. Finally, a key limitation is that we have no information on which parts of the abstract that lead the coders to make a coding decision. This makes the contribution of the abstract somewhat less precise. We do know, however, that the full text article has been consulted in cases where the coder was not able to make a decision from the abstract alone. We attempted to increase the vocabulary size to 10,000 words and observed some improvement to precision but typically at the expense of recall.

For experiment 3, classifying *Publication type*, we see that systematic reviews are much more likely to be both detected and classified correctly than non-systematic reviews; a reasonable explanation for this is that the former is much more likely to be explicitly named in the abstract than the latter, which is also a generic grouping category for “everything else”.

A similar observation can be made for experiment 4. As with the previous experiment, the *No data* class is likely to suffer from being implicit rather than explicit: from the reviewer’s point of view this label is applied in the absence rather than the presence of information.

A general source of error for all experiments is that the quality of the initial labeled publications is likely to fluctuate, especially for the earliest efforts. This is natural for any type of manual coding and classification project: it takes time for best practices to be established and knowledge to be disseminated among coders and the coding guide is likely to go through several revisions based on lessons learned during the coding process. Once more data is available this should become less of a problem.

When looking at ways to improve performance from a data point of view, an obvious activity would be to add additional training data. Since the evidence map project is still ongoing, additional coding data is being created and will provide a valuable basis for future experiments. Moreover, as the coders get more practice and experience the quality is likely to improve. Another possible effort is to improve the precision of the coding by having coders highlight the parts of the text that support their coding decision. This could improve the classifiers by allowing for more targeted training. Since the full publication text has also been used, integrating full text where possible—or just indicating when this is the case—could make a positive difference. It is also worth noting that the code book specification has been simplified since our initial data export, which should make future classification easier.

Comparing the performance of our results with that of similar research on automated classification of scientific literature is not straightforward but some observations can be made. For example, in [21] we see F-scores of around 0.50 which is in the same area as our experiment 2, which had the largest number of classes. This study had a much larger training set but it is difficult to compare the complexity of the tasks. Often there are strict requirements that a high level of recall must be sustained, such as e.g. 0.95 in [18]. We have left considerations of what the acceptable recall—and the subsequent effect on workload reduction—for our task is for future work. In [19] the best F1 score was almost 0.90 but again with a more training data to work with.

## Conclusions

We wanted to investigate if it was possible to use machine learning, specifically deep learning-based neural network models, to replicate a coding and classification scheme applied by expert coders over a perid of several weeks to COVID-19-related publications. Our experiments showed that even with the simplest possible text representations and generic neural network architectures it was possible to get promising results.

To improve results further a natural place to start would be experimenting with deep learning architectures and best-practices that are better geared towards text classification, not the least when it comes to making use of word context and embeddings rather than the simple one-hot encoding currently employed. We conducted one experiment using external pretrained embedding vectors but they did not provided any performance boost. Further experiments with more data are highly relevant. Also, for small data sets traditional approaches such as support-vector machines often exhibit comparable performance to neural nets and thus warrants a comparison. Since both the evidence map project and the ensuing research collaboration came about in a rush we hope to address these improvements in future work. The aim of this paper is not methodological novelty but rather to highlight the potential of a unique handcrafted dataset.

The long-term goal is to build classifiers that can be used as a basis for coding process and decision support, thereby reducing the time spent and effort needed by the coders. While high classifier performance is a key requirement, the importance of user interface should not be forgotten. This is a particular challenge for applications where machine learning is a key component. The suggested codings will never be perfect and it is therefore crucial to establish a coder workbench that allows for both approving, modifying and rejecting the automated suggestions while at the same time allowing for manual review and oversight. Given that the coding will be an ongoing process, finding ways to iteratively improve the classifiers would be of particular interest. The history of health-related decision support applications is both long and chequered—but with several lessons to learn from [27].

For our experiments in this paper a simple evaluation against the gold standard was sufficient. However, when applying the classifiers towards assisting the coding process different evaluation metrics must be considered, e.g. the time spent coding and changes to inter-coder agreement. Automated approaches are evaluated based on measurements of precision and recall but we know very little about what this means to those who wish to implement automation into workflows where a high-quality knowledge product is expected as the result. More knowledge is needed about acceptable error rates when applying decision-support technology to real-life needs and workflows.

While these initial experiments show promise for automated coding it is unlikely that the need for manual verification will be completely eliminated. Nonetheless, the amplification of highly skilled manual labor will improve quality, timeliness and frequency of updates by automating repetitive chores, new content detection, evidence integration, validation and consistency of results. Explainable artificial intelligence (AI) and explicit semantic reasoning in verifiable processes will allow experts to make predictable and trustable high-quality evidence maps. While we see immediate short-term potential in improving how knowledge is communicated for handling the COVID-19 crisis, the proposed technology can also have longer-term effects on medical information dissemination and management. As research communities become more advanced, global and specialised, the need for handling information flow and establishing best practices is unlikely to subside.

## Data Availability

The data we used is not available yet as the project where it was collected is still ongoing. Release of a more comprehensive dataset will be considered for future work.

## Abbreviations

AI: Artificial Intelligence
CORD-19: COVID-19 Open Research Dataset
JSON: JavaScript Object Notation
MeSH: Medical Subject Headings
NIPH: Norwegian Institute of Public Health
NLP: Natural language processing
NTNU: Norwegian University of Science and Technology
